# Growth of single crystalline films on lattice-mismatched substrates through 3D to 2D mode transition

**DOI:** 10.1038/s41598-020-61596-w

**Published:** 2020-03-13

**Authors:** Naho Itagaki, Yuta Nakamura, Ryota Narishige, Keigo Takeda, Kunihiro Kamataki, Kazunori Koga, Masaru Hori, Masaharu Shiratani

**Affiliations:** 1 Graduate School of Information Science and Electrical Engineering, Kyushu University, Fukuoka, 819-0395 Japan; 2 Department of Electrical and Electronic Engineering, Meijo University, Nagoya, 468-8502 Japan; 3 Graduate School of Engineering, Nagoya University, Nagoya, 464-8603 Japan

**Keywords:** Surfaces, interfaces and thin films, Electronic devices, Design, synthesis and processing

## Abstract

Regarding crystalline film growth on large lattice-mismatched substrates, there are two primary modes by which thin films grow on a crystal surface or interface. They are Volmer-Weber (VW: island formation) mode and Stranski-Krastanov (SK: layer-plus-island) mode. Since both growth modes end up in the formation of three-dimensional (3D) islands, fabrication of single crystalline films on lattice-mismatched substrates has been challenging. Here, we demonstrate another growth mode, where a buffer layer consisting of 3D islands initially forms and a relaxed two-dimensional (2D) layer subsequently grows on the buffer layer. This 3D-2D mode transition has been realized using impurities. We observed the 3D-2D mode transition for the case of ZnO film growth on 18%-lattice-mismatched sapphire substrates. First, nano-sized 3D islands grow with the help of nitrogen impurities. Then, the islands coalesce to form a 2D layer after cessation of the nitrogen supply, whereupon an increase in the surface energy may provide a driving force for the coalescence. Finally, the films grow in 2D mode, forming atomically flat terraces. We believe that our findings will offer new opportunities for high-quality film growth of a wide variety of materials that have no lattice-matched substrates.

## Introduction

Success in developing semiconductor devices has been limited thus far because of lattice-mismatch problems between growth layers and substrates. ZnO-based devices are such examples. ZnO is a multi-functional material with a wide range of existing and emerging applications^1,2^, such as transparent conducting electrodes^3–6^, surface-acoustic-wave (SAW) devices^7,8^, plasmonic devices^9^, thin film transistors^10–14^, resistance switching memristors^15^, gas sensors^16,17^, photo detectors^18,19^, and x-ray imaging systems^20^. Recently, ZnO has been recognized as a candidate for high-performance ultraviolet light emitting diodes (LED) and laser diodes (LD) because of its wide and direct bandgap of 3.37 eV^21,22^. The large exciton binding energy (60 meV) is an advantage over a commercial LED material, GaN (25 meV). The most common substrate for ZnO film growth is sapphire because of its high crystal quality and the availability of large size wafers. However, the large lattice mismatch between ZnO and sapphire (18%) results in considerable stress in ZnO films. Stress influences the dislocation density^23^, the surface morphology, and optical properties^24^ of thin films that have significant numbers of grain boundaries and/or crystal mosaics^25–27^.

**Figure 1:**
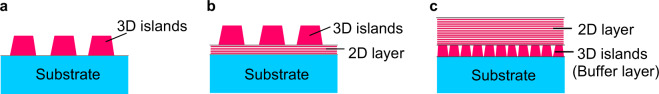
Schematic diagram of the three possible growth modes of heteroepitaxy on large lattice-mismatched substrates: (**a**) Volmer-Weber (VW: island formation) mode, (**b**) Stranski-Krastanov (SK: layer-plus-island) mode, and (**c**) island-plus-layer growth mode where a relaxed buffer layer consisting of 3D islands initially forms and a relaxed two-dimensional (2D) layer subsequently grows.

Regarding the heteroepitaxy of large lattice-mismatched systems, there are two primary modes by which thin films grow on a crystal surface or interface. They are i) Volmer-Weber (VW: island formation) mode^28^ and ii) Stranski-Krastanov (SK: layer-plus-island) mode (Fig. 1a,b)^29,30^. Since both growth modes end up in the formation of three-dimensional (3D) islands, fabrication of single crystalline films on lattice-mismatched substrates has been challenging^28–30^.

Here, we demonstrate another growth mode, where a relaxed buffer layer consisting of 3D islands initially forms and relaxed two-dimensional (2D) layers subsequently grow on the buffer layer (island-plus-layer growth mode) (Fig. 1c). We describe the difference between the SK mode and the island-plus-layer growth mode. In SK mode, a highly strained 2D layer initially forms, taking advantage of the low surface energy compared to that of 3D islands. As the 2D layer grows, however, the total free energy increases with increasing film thickness because the elastic energy stored in the system increases. Finally, at a critical thickness, a 2D to 3D growth mode transition occurs such that the lattice strain is relaxed and thus the total free energy is minimized. That is, the 2D to 3D mode transition occurs when the elastic energy cost of the 2D growth exceeds the surface energy cost of islanding. In contrast, in the island-plus-layer growth mode, a buffer layer consisting of densely packed nano-sized 3D islands initially grows. Here, the cost of islanding is reduced by insoluble impurities that are adsorbed on the surface^31–34^. Since such small islands with large surface-to-volume ratios relieve the elastic strain effectively at the surface^35,36^, the islands have good in-plane and out-of-plane alignment of the crystal axis. After the lattice strain is relaxed, we desorb the impurity atoms from the surface, which leads to the coalescence of the 3D islands (2D layer formation). This transition from the 3D to 2D growth mode occurs only when the lattice planes of initially formed 3D islands in the buffer layer are well aligned with those of the substrates; otherwise, coalescence of islands does not occur. The possibility of this 3D-2D mode transition has been theoretically predicted by Tinjod *et al*., who presented a simple equilibrium model, taking into account not only the lattice mismatch, but also the dislocation formation energy and the surface energy^37^.

Here, we experimentally demonstrate the 3D-2D mode transition for the case of ZnO film growth on 18%-lattice-mismatched sapphire substrates, where nitrogen atoms are employed as impurities. Not only the roles of impurities but also other key parameters to realize the 3D-2D mode transition are discussed.

## Results

### 3D-2D mode transition during ZnO growth on 18%-lattice-mismatched sapphire substrates

**Figure 2:**
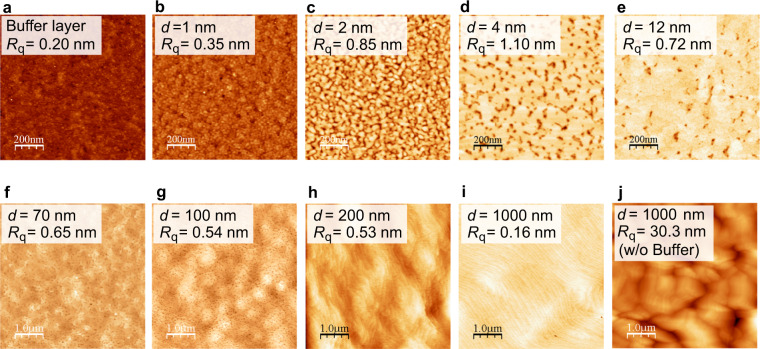
AFM images of ZnO films fabricated on 18%-lattice-mismatched sapphire substrates. (**a–i**) AFM images showing the evolution of surface morphology upon deposition of (**a**) 10-nm-thick buffer layer and (**b–i**) 1–1000 nm of ZnO films deposited on the buffer layer. Here, the buffer layer was fabricated with N_2_ gas, while 1–1000 nm-thick ZnO films were fabricated without N_2_ gas on the buffer layer. (**j**) AFM image of a 1000-nm-thick ZnO film fabricated without a buffer layer. No post-deposition annealing was performed for any samples.

We observed the 3D-2D mode transition during ZnO film growth on 18%-lattice-mismatched sapphire substrates. AFM images in Fig. 2 show the evolution of surface morphology upon deposition of a 10-nm-thick buffer layer and 1–1000 nm of ZnO films deposited on the buffer layer. We first deposited 10-nm-thick buffer layers with N_2_ gas at 735 °C, then ceased supplying N_2_ gas, and deposited 1–1000 nm-thick ZnO films without N_2_ gas on the buffer layers. No post-deposition annealing was performed. For comparison, an AFM image of a 1000-nm-thick ZnO film fabricated without a buffer layer is shown in Fig. 2j. As shown in Fig. 2a, the buffer layer fabricated with N_2_ consists of densely packed nano-sized 3D islands. After cessation of the N_2_ gas supply, crystal grains originating from each island grow in the buffer layers, and they coalesce to form 2D layers (Fig. 2b–e). Eventually, ZnO films grow in 2D mode, where the films have an atomically flat surface with steps of 0.26 nm in height (Fig. 2h,i). This height corresponds to half of the c-axis length of ZnO. By contrast, in the case without N_2_, the growth of ZnO film ended up in the formation of 3D structures as shown in Fig. 2j, and the root-mean-square (RMS) roughness (*R*_*q*_) of the surface is significantly large at 30 nm. These results clearly show that nitrogen plays a key role in determining the growth mode of ZnO films on sapphire substrates, and we discuss the mechanism later.

**Figure 3:**
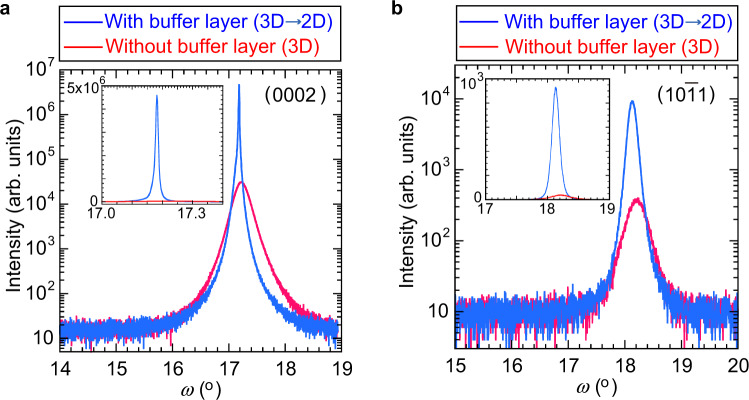
Rocking curves of 1000-nm-thick ZnO films fabricated on sapphire substrates: (**a**) symmetric (0002) reflections, and (**b**) asymmetric ($10\bar{1}1$) reflections. The blue curves are for ZnO film on the buffer layer fabricated with N_2_ gas. The red curves are for ZnO film fabricated without a buffer layer. The insets show the curves plotted on a linear scale.

**Figure 4:**
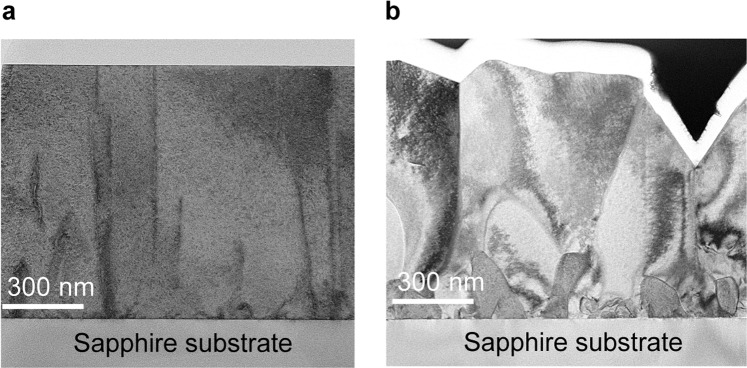
Bright field TEM images of ZnO films grown on sapphire substrates (**a**) with the buffer layer fabricated using N_2_ gas and (**b**) without the buffer layer.

High crystal quality of ZnO films grown through the 3D-2D mode transition was proved by x-ray diffraction analysis. Figure 3 shows the rocking curves around the symmetric (0002) and asymmetric ($10\bar{1}1$) reflections of the 1000-nm-thick ZnO film on the buffer layer fabricated with N_2_. The inset shows the linear plots. For comparison, rocking curves of a 1000-nm-thick ZnO film fabricated without a buffer layer are shown. For the ZnO film on the buffer layer (3D** →** 2D), the full width at half maximum (FWHM) of the (0002) and ($10\bar{1}1$) planes are 0.01° and 0.09°, respectively. These values are significantly small compared with those of the ZnO film grown without a buffer layer, where the FWHM of the (0002) and ($10\bar{1}1$) planes are 0.25° and 0.35°, respectively. The edge-type threading dislocation densities are estimated using the relation, ${{N}}_{{E}}={{\beta }_{{E}}}^{2}/(4.36\times |{{\rm{b}}}_{{E}}{|}^{2})$, where ${{\boldsymbol{\beta }}}_{{\boldsymbol{E}}}$ is the FWHM value of the rocking curve for the (0002) plane, and $|{{\rm{b}}}_{{E}}|$ is the length of the Burgers vectors for the edge component $(|{{\rm{b}}}_{{E}}|=11\bar{2}0/3=0.1083\,{\rm{nm}})$^38–40^. The estimated edge-type dislocation densities of the films grown with and without the buffer layer are 6.0 × 10^7^ cm^−2^ and 3.7 × 10^10^ cm^−2^, respectively. These results indicate that ZnO films grown through the 3D-2D mode transition possess high crystal quality with low defect density as well as good in-plane and out-of-plane alignment of the crystal axis. Transmission electron microscopy (TEM) measurements also reveal the high crystal quality of the ZnO film grown through the 3D-2D mode transition. Figure 4 shows the bright field TEM images of ZnO films on sapphire substrates. The film grown on the buffer layer fabricated with N_2_ (3D** →** 2D) is a single crystal with a significantly flat surface, whereas the film fabricated without a buffer layer possesses a polycrystalline structure with a rough surface. In addition, we found that the electrical properties are improved by using the buffer layer. All the films prepared in this study exhibit n-type behaviour; however, the films grown on the buffer layer (3D** →** 2D) have low residual carrier density (2 × 10^17^ cm^−3^) and high carrier mobility (90 cm^2^/Vs), one order of magnitude lower and 1.5 times higher than the film fabricated without a buffer layer, respectively.

### Role of impurity

**Figure 5:**
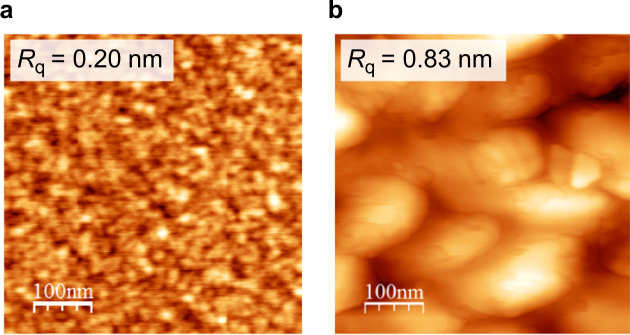
AFM images of 10-nm-thick buffer layers fabricated on sapphire substrates (**a**) with N_2_ and (**b**) without N_2_.

We now discuss the roles of impurities that lead to the 3D-2D mode transition of ZnO film growth on sapphire substrates. Figure 5 shows AFM images of 10-nm-thick buffer layers fabricated with and without N_2_. Significant changes in the grain size and in the surface roughness are observed. The lateral island size of the film deposited with N_2_ is about 10 nm, one order of magnitude smaller than that of the film deposited without N_2_. Since N_2_ molecules themselves are chemically stable (not active), we consider that N atoms play important roles in determining the island size. As we described elsewhere, N atoms in low-pressure plasmas are mainly generated by electron-impact processes^41^. These processes consist of dissociative collisions between an electron and a N_2_ molecule and dissociative collisions between an electron and a N_2_ molecular ion:

A $${\rm{e}}+{{\rm{N}}}_{2}\to {\rm{N}}+{\rm{N}}+{\rm{e}},$$

B $${\rm{e}}+{{\rm{N}}}_{2}^{+}\to {\rm{N}}+{\rm{N}}.$$

Reaction B occurs through an ionization collision between an electron and a N_2_ molecule:

C $${\rm{e}}+{{\rm{N}}}_{2}\to {{\rm{N}}}_{2}++2{\rm{e}}.$$

In pure N_2_ plasmas, the dominant process for N-atom production is reaction A because of the large rate coefficient^42^. For example, at the electron temperature of 2 eV in a plasma where the electron energy distribution functions are Maxwellian, the rate coefficients of reaction A and reaction C are about 5 × 10^−11^ and 5 × 10^−12^ cm^−3^s^−1^, respectively^43^. In Ar-N_2_ plasmas, there is another contribution to N-atom production: charge exchange between the N_2_ molecule and Ar ion:

D $${{\rm{Ar}}}^{+}+{{\rm{N}}}_{2}\to {\rm{Ar}}+{{\rm{N}}}_{2}^{+}$$

which is followed by reaction B that has a large rate coefficient of 2 × 10^7^ cm^−3^s^−1^ ^43^.

In conclusion, the dissociation ratio [N]/[N_2_] is about 10^−3^ in our Ar-N_2_ magnetron plasma with an electron temperature of 3 eV^44^. That is, the N-atom density in the plasma is of the order of 10^10^ cm^−3^, which was confirmed by means of vacuum ultraviolet absorption spectroscopy (VUVAS)^45,46^. This value is comparable to the O-atom density in the plasma originating from ZnO sputtering targets. In contrast, the concentration of N-atoms incorporated into the ZnO film is significantly small at 10^17–18^ cm^−3^ (see Supplementary Fig. S1), which is 4–5 orders of magnitude lower than the oxygen concentration in the film. This low nitrogen concentration in the film is due to the low solubility in ZnO^27,47,48^, and this property of nitrogen is what we consider to lead to grain size decrease. That is, the low solubility makes N-atoms segregate on the surface as well as at the grain boundaries, and thus lower the surface free energy per unit area^31–34^. In fact, we observed that nitrogen species are segregated on the film surface by measuring the depth profile of nitrogen concentration of the buffer layer with secondary ion mass spectroscopy (SIMS) (see Supplementary Fig. S1). As a result, in the buffer layer, relaxed small islands form, instead of strained large islands, because the gain of elastic relaxation energy overcompensates for the cost due to extra surface energy (a smaller island has a higher surface-to-volume ratio). After cessation of the N-atom supply, however, the surface energy drastically increases. This increase in the surface energy may provide a driving force for the coalescence of 3D islands, and consequently causes 3D to 2D mode transition as described above. By contrast, in the case without N_2_, it is hard to form 3D islands as small as the islands fabricated with N_2_, because the cost of increased surface energy exceeds the gain of elastic energy.

**Figure 6:**
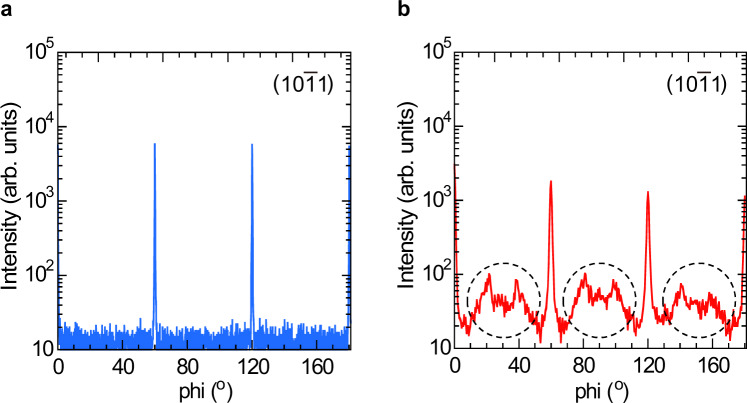
XRD phi scan profiles of the asymmetric ($10\bar{1}1$) reflection of the buffer layers deposited (**a**) with N_2_ and (**b**) without N_2_. Diffraction signals due to rotated domains are encircled by dotted lines.

Next, we examine effects of nitrogen on the crystal quality of the buffer layer itself. Figure 6 shows XRD phi scans of the asymmetric ($10\bar{1}1$) reflection of the buffer layer deposited with and without N_2_, where the diffraction intensity is plotted on a logarithmic scale. In both cases, peaks at 60° with respect to each other are observed, indicating the 3D islands in the buffer layers have six-fold symmetry of the hexagonal lattice. This is evidence of epitaxial growth of the 3D islands on the sapphire substrates, where the epitaxial relationship between ZnO and sapphire is [0001]_ZnO_ | |[0001]_sapphire_ and [$10\bar{1}0$] _ZnO_ | |[$11\bar{2}0$]_sapphire_. There is, however, a significant difference in the crystal quality between buffer layers fabricated with and without N_2_. The FWHM of the phi scans for the buffer layer fabricated with N_2_ is small at 0.33°, suggesting that the islands have small twist angles. This value is about one-fourth of the buffer layer fabricated without N_2_. Furthermore, no rotated domains are observed for the case with N_2_, whereas a small but significant amount of rotated domains are observed for the case without N_2_ (encircled by a dotted line in Fig. 6b). This difference in the in-plane alignment is explained as the result of the difference in the island size. In the small islands fabricated with N_2_, the strain relaxation occurs efficiently at the surface of the 3D islands^36,49^. In contrast, the lattice strain in the large islands fabricated without N_2_ is not fully released through island formation because of the low surface-to-volume ratios. Thus, a large amount of misfit dislocations form in the islands to gain elastic relaxation energy, leading to somewhat poor crystal-axis alignment.

This hypothesis is consistent with the above-mentioned observation of the crystal quality of subsequently grown 1000-nm-thick ZnO films, where the edge-type dislocation density of ZnO films grown on the buffer layer fabricated with N_2_ is three orders of magnitude lower than that of the film fabricated without the buffer layer. We consider that the good in-plane alignment along with the smooth surface of the buffer layer fabricated with N_2_ promote the above-mentioned coalescence of 3D islands of the subsequently grown layers.

### Key parameters of 3D-2D mode transition

**Figure 7:**
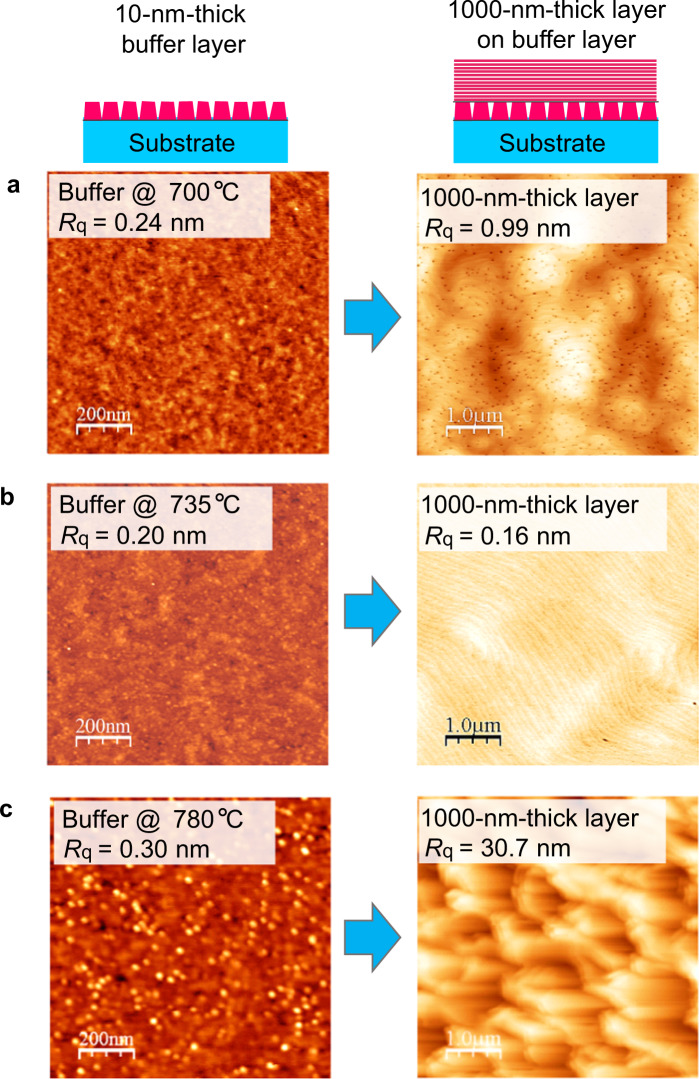
AFM images of 10-nm-thick buffer layers and subsequently grown 1000-nm-thick layers on the buffer layers. The deposition temperatures of the buffer layers were (**a**) 700 °C, (**b**) 735 °C, and (**c**) 780 °C. All 1000-nm-thick layers were fabricated at 700 °C.

**Figure 8:**
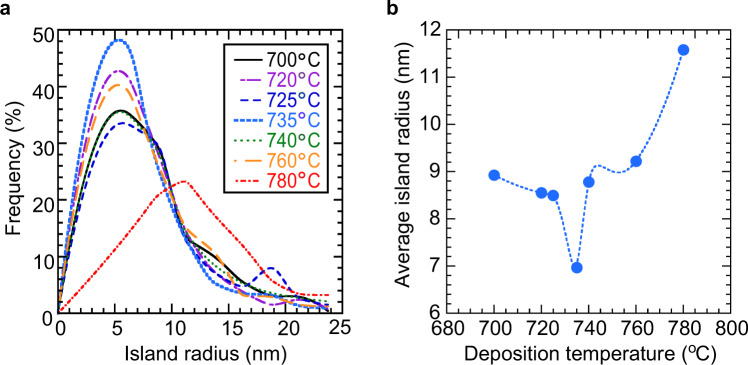
The island radius of 10-nm-thick buffer layers, derived from AFM images. (**a**) The distributions of the island radius for various deposition temperatures. (**b**) The average island radius as a function of the deposition temperature.

As mentioned above, the size of the 3D islands and the surface smoothness seem to be the keys for growth of high-quality films through the 3D-2D mode transition. Here, we discuss the impact of these parameters of the buffer layers on the subsequently grown 1000-nm-thick layers. Figure 7 shows AFM images of 10-nm-thick buffer layers fabricated at 700–780 °C and subsequently grown 1000-nm-thick layers on the buffer layers. All 1000-nm-thick ZnO films were fabricated at 700 °C. The deposition temperature of the buffer layer apparently influences the surface morphology of subsequently grown 1000-nm-thick ZnO films, and we consider that the size of the 3D island is one of the keys. Figure 8 shows the distribution of the island radius and the average radius of the buffer layers, derived from AFM images, as a parameter of deposition temperature. A very sharp drop in the average radius is observed at 735 °C, where the subsequently grown 1000-nm-thick ZnO film has an atomically flat surface with straight step edges as shown in Fig. 7b (right). This is the result of small islands having large surface-to-volume ratios, bring about efficient release of strain energy at the grain surfaces. The buffer layer fabricated at 700 °C has islands with an average radius of 9 nm, 1.3 times larger than that of the buffer layer fabricated at 735 °C. On this buffer layer, the ZnO film grows in 2D mode but has curved-step edges and a large number of pits, attributed to the residual strain in the film.

**Figure 9:**
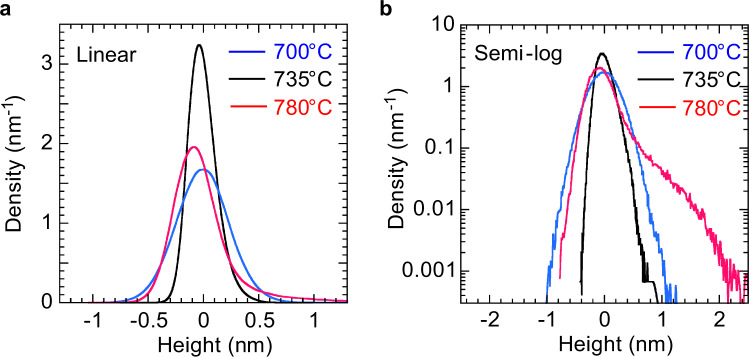
Surface height distribution of 10-nm-thick buffer layers fabricated at 700–780 °C. (**a**) Linear plots. (**b**) Semi-log plots.

Surface smoothness, characterized by surface height distribution, of the buffer layers is another key for the 3D-2D mode transition. For example, if we compare the buffer layers fabricated at 700 °C and 780 °C, there is no significant difference in RMS roughness (*R*_q_) (Fig. 7a,c, left). However, the morphologies of the subsequently grown 1000-nm-thick ZnO films are quite different. ZnO film on the buffer layer fabricated at 700 °C grew in 2D mode, where the surface has step-terrace structures with RMS roughness of 0.99 nm (Fig. 7a, right), while ZnO film on the buffer layer fabricated at 780 °C grew in 3D mode, where the film has a rough surface with RMS roughness of 30.7 nm (Fig. 7c, right). This is attributed to the difference not only in the island size but also in the surface smoothness that is not just defined by the RMS roughness of the buffer layers. Figure 9 shows the surface height distribution, derived from AFM images, of buffer layers fabricated at 700–780 °C. For the buffer layer fabricated at 780 °C, a tail appears around *z* = 1–2 nm in the distribution (Fig. 9b), yet such a tail is not clearly observed when the distribution is plotted on a linear scale (Fig. 9a). This result indicates that the surface has some spikes that limit the migration of adatoms and/or lead to secondary nucleation, resulting in the subsequent growth to be not in 2D mode but in 3D mode.

In addition, we consider that the morphological changes of buffer layers with the deposition temperature are brought about by the change in the nitrogen coverage on the surface. In fact, we observed that the deposition rate of buffer layers increases from 0.18 to 0.22 nm/s as the deposition temperature increases from 700 to 780 °C^50^. This change in the deposition rate indicates the change in the coverage of N-atoms on the surface because the adsorbed but non-soluble impurities should reduce the supply of sputtered species on the surface^31,50^. A more detailed discussion will appear elsewhere.

Finally, we briefly address the differences between our buffer layers fabricated with impurities and low-temperature (LT) buffer layers developed for GaN growth on 15%-lattice-mismatched sapphire substrates^51,52^. Their major roles are essentially the same: promotion of lateral growth of the subsequent layers, and suppression of dislocations in the layers. However, we found some differences in the details, and here we discuss two of them.

The LT buffer layers for GaN consist of fine crystallites embedded in an amorphous matrix and the morphology is obtained by controlling the growth temperature^51,52^. The fine crystallites have the same orientation as the substrate and serve as nucleation centres for the subsequently grown GaN film. On the buffer layer, GaN crystals that originate from the fine crystallites grow laterally, owing to the low interfacial free energy between GaN crystals and the buffer layer, and finally coalesce to form a single crystal film. That is, the roles of the LT-buffer layer are to supply the nucleation sites with the same crystal orientation as the substrate, and to realize low interfacial free energy between GaN and the substrates, leading to lateral growth of GaN crystals on the buffer layer. In our case, on the other hand, buffer layers consist of densely packed nano-sized grains that have the same orientation as the substrate. Our nano-sized grains that have a high surface-to-volume ratio relieve the strain efficiently at the surface, resulting in the low dislocation density. On the buffer layer, relaxed crystals grow originating from each grain in the buffer layers, and they coalesce to form a 2D layer due to the low interfacial energy between the 2D layers and the buffer layers. Here, we consider that a drastic increase in the surface energy provides the driving force for the coalescence of crystal grains. During buffer layer formation, the surface energy is lowered by adsorbed impurities, and this is why nano-sized grains are obtained. After cessation of the impurity supply, the surface energy increases due to desorption of impurities, providing a driving force for coalescence of crystal grains.

Another difference from the LT-buffer layer for GaN growth is in the methodology to control the film morphology. For the case of LT-buffer layers, the tuning nob adjusts the deposition temperature, whereas we control the film morphology through manipulating the adsorption/desorption behaviours of impurities. We consider that our method works well especially for materials like ZnO that are easily crystallized even at room temperature.

In summary, by manipulating the surface energy through adsorption/desorption behaviours of N-atoms, single crystalline ZnO films are grown on 18%-lattice-mismatched substrates via 3D-2D mode transition. Here, buffer layers consisting of nano-sized 3D islands initially grow with the help of impurities. After cessation of the impurity supply, the islands rapidly coalesce to form a 2D layer, where an increase in the surface energy provides a driving force for coalescence. Finally, the films grow in 2D mode and have an atomically flat surface. There are two important parameters for the growth of high-quality films through the 3D-2D mode transition: the size of the 3D island and the surface height distribution of the 3D island layer. In small islands, having large surface-to-volume ratios, the strain relaxation occurs at the surface of the 3D islands. As a result, in-plane aligned 3D islands with low defect density are grown. Such 3D islands lead to the coalescence of 3D islands of the subsequently grown layers if the 3D layer has a smooth surface promoting the adatom migration. We believe that our findings on this new growth mode will open up a new pathway for high-quality film growth of a wide variety of materials that have no lattice-matched substrates.

## Methods

All films were fabricated by radio frequency (RF) magnetron sputtering. First, 10-nm-thick buffer layers were fabricated with N_2_ gas on c-plane sapphire substrates at 700–735 °C. ZnO ceramic targets (2 inch, >99.99 purity) were used, and the supplied RF power was 100 W. The used gases were Ar and N_2_, and the flow rates were 24 and 1 sccm, respectively. The total gas pressure was 0.35 Pa. Vacuum ultraviolet absorption spectroscopy (VUVAS) was performed during the deposition to monitor the absolute value of N-atoms and O-atoms in the sputtering plasma. The VUVAS system was equipped with a micro discharge hollow–cathode lamp, and the VUV light passing through the sputtering plasma was detected by a VUV monochromator and a photomultiplier tube^45,46^. Plasma parameters were measured by a Langmuir probe set at 50 mm above the cathode, where the electron density and the electron temperature were about 6 × 10^9^ cm^−3^ and 3 eV, respectively^44^. The thickness of the buffer layers was confirmed by x-ray reflectometry. The nitrogen concentration of the films was measured by secondary ion mass spectroscopy (SIMS).

Next, 1000-nm-thick ZnO films were deposited on the 10-nm-thick buffer layers. Here, no intentional introduction of impurities into the deposition atmosphere was performed. The substrate temperature was 700 °C. Ar and O_2_ were used as sputtering gases, and the flow rates were 45 and 5 sccm, respectively. The total gas pressure was 0.70 Pa. Two ZnO ceramic targets (2 inch, >99.99 purity) were used and the RF power supplied to the cathodes was 60 W. The thickness of ZnO films was confirmed by scanning electron microscopy. No post-deposition annealing was performed.

The crystal quality was evaluated by x-ray diffraction (XRD) using a four-circle texture diffractometer and Cu Kα 1 radiation. Transmission electron microscopy (TEM) was done with a JEOL JEM-ARM200F using cross-sectional preparation. The surface morphologies were measured by tapping mode atomic force microscopy (AFM). Electrical properties were evaluated by Hall-effect measurements using the four-point van der Pauw configuration at room temperature.

## Supplementary information

Supplementary Information.
