# Correction: Growth of single crystalline films on lattice-mismatched substrates through 3D to 2D mode transition

**DOI:** 10.1038/s41598-026-36788-5

**Published:** 2026-01-23

**Authors:** Naho Itagaki, Yuta Nakamura, Ryota Narishige, Keigo Takeda, Kunihiro Kamataki, Kazunori Koga, Masaru Hori, Masaharu Shiratani

**Affiliations:** 1 Graduate School of Information Science and Electrical Engineering, Kyushu University, Fukuoka, 819-0395 Japan; 2 Department of Electrical and Electronic Engineering, Meijo University, Nagoya, 468-8502 Japan; 3 Graduate School of Engineering, Nagoya University, Nagoya, 464-8603 Japan

Correction to: *Scientific Reports* 10.1038/s41598-020-61596-w, published online 13 March 2020

This Article contains an error in the Results section, where a crystallographic plane is incorrectly written in the description of the X-ray rocking curve analysis. As a result,

“The edge-type threading dislocation densities are estimated using the relation, *NE*=*βE24.36*×*|*b*E|2*, where *βE* is the FWHM value of the rocking curve for the (0002) plane, and *|*b*E|* is the length of the Burgers vectors for the edge component *(*b*E*=*11203*=*0.1083*nm*)*.”

should read:

“The edge-type threading dislocation densities are estimated using the relation, *NE*=*βE24.36*×*|*b*E|2*, where *βE* is the FWHM value of the rocking curve for the (10-11) plane, and *|*b*E|* is the length of the Burgers vectors for the edge component *(*b*E*=*11203*=*0.1083*nm*)*.”

