# Supplementary material for: Growth of single crystalline films on lattice-mismatched substrates through 3D to 2D mode transition
Source: Sci Rep. 2020 Mar 13;10:4669. doi: 10.1038/s41598-020-61596-w (PMC7070095; doi:10.1038/s41598-020-61596-w)
Supplement: Supplementary file 1 — Supplementary Information. [file 41598_2020_61596_MOESM1_ESM.pdf]

Supplementary Information

Growth of single crystalline films on lattice-mismatched substrates through 3D to 2D mode transition

Naho Itagaki[1*], Yuta Nakamura[1], Ryota Narishige[1], Keigo Takeda[2], Kunihiro Kamataki[1], Kazunori Koga[1], Masaru Hori[3], and Masaharu Shiratani[1]

[1]Graduate School of Information Science and Electrical Engineering, Kyushu University, Motooka, Fukuoka 819-0395, Japan
[2]Department of Electrical and Electronic Engineering, Meijo University, Tempaku, Nagoya, 468-8502, Japan
[3]Graduate School of Engineering, Nagoya University, Chikusa, Nagoya 464-8603, Japan
[*]itagaki@ed.kyushu-u.ac.jp

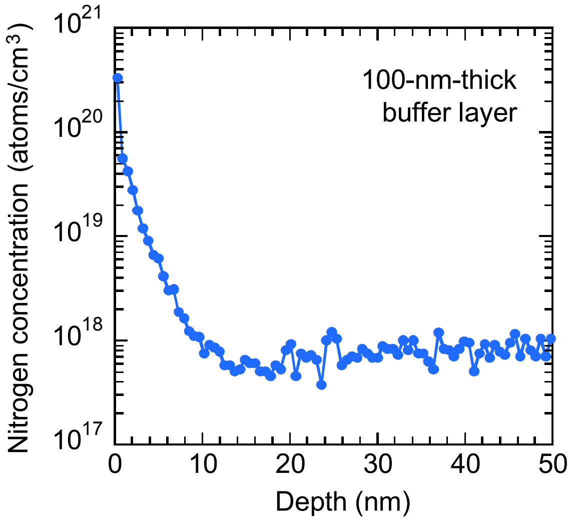

Figure S1. The depth profile of nitrogen concentration of a buffer layer, measured by secondary ion mass spectroscopy (SIMS). Here the film thickness is 100 nm. The segregation width is less than 10 nm, taking into account of knock-on effect in which atoms of the sample are driven deeper into the sample during the sputtering process by the primary ion beam used for analysis.
